# Role of Heat Shock Protein 47 in Transdifferentiation of Human Tenon's Fibroblasts to Myofibroblasts

**DOI:** 10.1186/1471-2415-12-49

**Published:** 2012-09-11

**Authors:** Samin Hong, Kyoungsoo Park, Jin Hyoung Kim, Sueng-Han Han, Jong Bok Lee, Gong Je Seong

**Affiliations:** 1 Institute of Vision Research, Department of Ophthalmology, Yonsei University College of Medicine, 50 Yonsei-ro, Seodaemun-gu, Seoul, 120-752, Republic of Korea; 2 Siloam Eye Hospital, 181 Deungchon-ro, Gangseo-gu, Seoul, 156-032, Republic of Korea

**Keywords:** Fibroblast, Fibrosis, Heat shock protein, Myofibroblast, Transforming growth factor-β

## Abstract

**Background:**

Heat shock protein 47 (Hsp47) is a well-known molecular chaperone in collagen synthesis and maturation. The aim of this study is to investigate its putative role in the transdifferentiation of Tenon’s fibroblasts to myofibroblasts.

**Methods:**

Primary cultured human Tenon’s fibroblasts were exposed to transforming growth factor-β1 (TGF-β1) for up to 48 hours. The mRNA levels of Hsp47 and α smooth muscle actin (αSMA) were determined by quantitative real time RT-PCR. After delivery of small interfering RNA (siRNA) molecules targeting Hsp47 into the cells, the expression of Hsp47 and αSMA proteins was determined by western immunoblotting.

**Results:**

TGF-β1 increased the mRNA expressions of both Hsp47 and αSMA in human Tenon’s fibroblasts, as determined by quantitative real time RT-PCR. However, it induced the protein expression of only αSMA but not Hsp47, as determined by western immunoblots. When siRNAs specific for Hsp47 were introduced into those cells, the TGF-β1-induced expression of αSMA was significantly attenuated on western immunoblots; after 48 hours of exposure to TGF-β1, the relative densities of immunobands were 11.58 for the TGF-β1 only group and 2.75 for the siRNA treatment group, compared with the no treatment control group (p < 0.001).

**Conclusions:**

Our data suggest that Hsp47 may be related to the TGF-β1-induced transdifferentiation of human Tenon’s fibroblasts to myofibroblasts.

## Background

Excessive subconjunctival fibrosis is a major cause of failure after glaucoma filtering surgeries [1]. Although transforming growth factor-β (TGF-β) is known to play a crucial role in this pathologic process and to induce the transdifferentiation of subconjunctival fibroblasts to myofibroblasts [2-5], the precise molecular mechanisms involved are not fully understood.

Heat shock protein 47 (Hsp47), also known as serpinh1 and collagen binding protein 1, is a member of the serpin superfamily of serine protease inhibitors and a molecular chaperone specific for procollagen [6-11]. It localizes to the endoplasmic reticulum (ER) and is involved in the post-translational modification of procollagen and maturation of collagen. Although a number of studies have found overexpression of Hsp47 in extraocular tissues in various fibroblastic diseases [12-21], only a few studies have investigated Hsp47 expression in ocular tissue [22-24].

In the present study, we assessed whether TGF-β increases Hsp47 expression in primary cultured human Tenon’s fibroblasts and whether Hsp47 is associated with the TGF-β-induced expression of α smooth muscle actin (αSMA), a phenotypic hallmark of the transdifferentiation of fibroblasts to myofibroblasts.

## Materials and methods

### Cell culture and exposure to TGF-β1

After obtaining approval from the Institutional Review Board of our institution, subjects who had no ocular/systemic disease except for strabismus received comprehensive information and provided written informed consent. All protocols were conducted in compliance with the tenets of the Declaration of Helsinki. Small Tenon’s capsule specimens were excised during strabismus surgeries and fibroblasts were isolated as previously described [5]. Cells were incubated in Dulbecco’s modified Eagle’s medium (DMEM; Life Technologies, Carlsbad, CA, USA) supplemented with 10% heat-inactivated fetal bovine serum (FBS; Life Technologies), 100 units/mL penicillin and 100 μg/mL streptomycin (Life Technologies) at 37°C and 5% CO_2_. We used cells between the third and fifth passages for this study, and cultures were allowed to reach about 80% confluence.

After 12 hours of serum starvation in serum-free media, fibroblasts were exposed to 5 ng/mL recombinant human TGF-β1 (R&D Systems, Inc., Minneapolis, MN, USA) for up to 48 hours. For the no treatment control group, the same volume of DMEM was added to the culture media instead of TGF-β1. All experiments were performed in quadruplicate at least, and repeated at least four times using independent cell cultures.

### RNA interference assay

Small interfering RNA (siRNA) molecules targeting Hsp47 mRNA were purchased from Santa Cruz Biotechnology, Inc. (Santa Cruz, CA, USA) and delivered into cells according to the manufacturer’s instructions. Briefly, the cells were seeded in 6-well culture plates at ~1.5 x 10^5^ cells per well in antibiotic-free DMEM. The 50 nM siRNA duplex was mixed with 2 μg/mL Lipofectamine^TM^ 2000 (Life Technologies) to allow the formation of transfection complexes. This mixture was then dispensed onto the cells and incubated for 16 hours at 37°C in a CO_2_ incubator. For negative control, the scrambled siRNAs were used instead of the siRNAs specific for Hsp47.

### Real time RT-PCR

Total RNA was extracted using RNeasy Mini Kit (QIAGEN, Venlo, Netherlands) and treated with DNase (QIAGEN) to remove contaminating DNA according to the manufacturer's directions. cDNA was synthesized from 2 μg of total RNA using SuperScript^TM^ III First-Strand Synthesis System for RT-PCR (Life Technologies).

Real time RT-PCR was performed with 50 ng cDNA per reaction using 25 μL of iQ SYBR® Green Supermix (Bio-Rad Laboratories, Hercules, CA, USA) containing 500 nM specific upstream and downstream primers (Table 1) in the iCycler iQ^TM^ Real-Time PCR Detection System (Bio-Rad Laboratories). The SYBR green data and level of target mRNA were analyzed by iCycler iQ^TM^ software with a relative ratio to β-actin of no treatment control.

**Table 1:** Primer sequence for real time RT-PCR

**Gene Name**	**Type**	**Sequence**
Hsp47	Forward	5’-CGC CAT GTT CTT CAA GCC A-3’
	Reverse	5’-CAT GAA GCC ACG GTT GTC C-3’
αSMA	Forward	5’-GTG TTA TGT AGC TCT GGA CTT TGA AAA-3’
	Reverse	5’-GGC AGC GGA AAC GTT CAT T-3’
β-actin	Forward	5’-GCG GGA AAT CGT GCG TGA CAT T-3’
	Reverse	5’-GAT GGA GTT GAA GGT AGT TTC GTG-3’

αSMA = α smooth muscle actin; Hsp47 = heat shock protein 47.

### Western immunoblots

Whole cellular proteins were extracted from primary cultured human Tenon’s fibroblasts. Briefly, total cell lysates were obtained using cell lysis buffer (Sigma-Aldrich, St. Louis, MO, USA) on ice for 10 minutes. The lysates were sonicated and the cell homogenates were centrifuged at 15,000 g for 10 minutes at 4°C.

Protein concentrations in the resultant supernatants were determined with the Bio-Rad Protein Assay (Bio-Rad Laboratories) based on the Bradford dye-binding procedure. Equal amounts of protein (10 μg) were boiled in Laemmli sample buffer and resolved by 7.5 or 12.0% sodium dodecyl sulfate polyacrylamide gel electrophoresis (SDS-PAGE). The proteins were transferred to polyvinylidene fluoride (PVDF) membranes and probed overnight with primary antibodies against human Hsp47, αSMA, and β-actin (diluted 1:500; Santa Cruz Biotechnology).

Immunoreactive bands were detected with horseradish peroxidase-conjugated secondary antibodies (diluted 1:2,000; Santa Cruz Biotechnology) and visualized by an enhanced chemiluminescent system (SuperSignal West Pico Chemiluminescent Substrates, Pierce Biotechnology, Inc., Rockford, IL, USA) on autoradiograph films.

### Statistical analysis

Data are expressed as means ± standard error of the mean (S.E.M.). Data were compared using Kruskal-Wallis one-way analysis of variance using the MedCalc program for Windows, version 11.4.2.0 (MedCalc Software bvba, Mariakerke, Belgium). P-values less than 0.05 were considered statistically significant.

## Results

**Figure 1:**
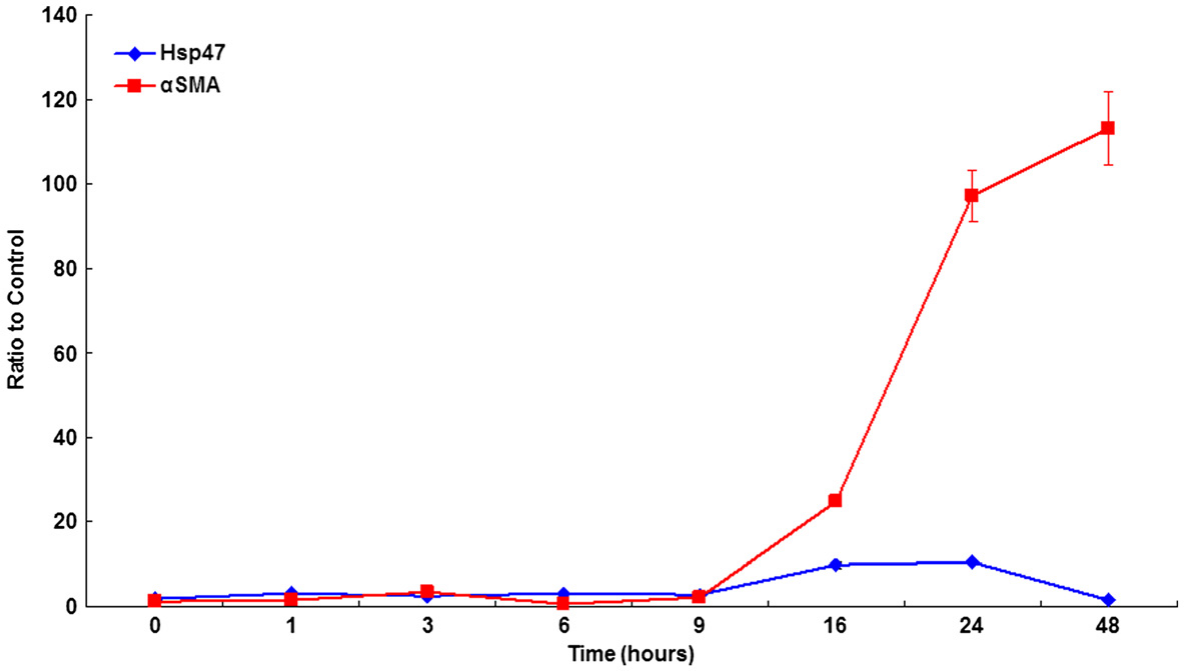
**Quantitative real time RT-PCR for heat shock protein 47 (Hsp47) and α smooth muscle actin (αSMA) after exposure to transforming growth factor-β1 (TGF-β1) for up to 48 hours.** The level of target mRNA was calculated using a relative ratio to β-actin of no treatment control and expressed as the mean ± S.E.M. (n = 16 for each group).

Quantitative data of real time RT-PCR for Hsp47 and αSMA are shown in Figure 1. In human Tenon’s fibroblasts, the mRNA levels of Hsp47 and αSMA were significantly increased at 16 hours after exposure to TGF-β1. At that time point, the ratios relative to the control were 9.822 ± 0.821 for Hsp47 and 24.910 ± 1.126 for αSMA. The mRNA level of αSMA was dramatically increased as time passed, but that of Hsp47 was returned to baseline after 48 hours exposure to TGF-β1.

**Figure 2:**
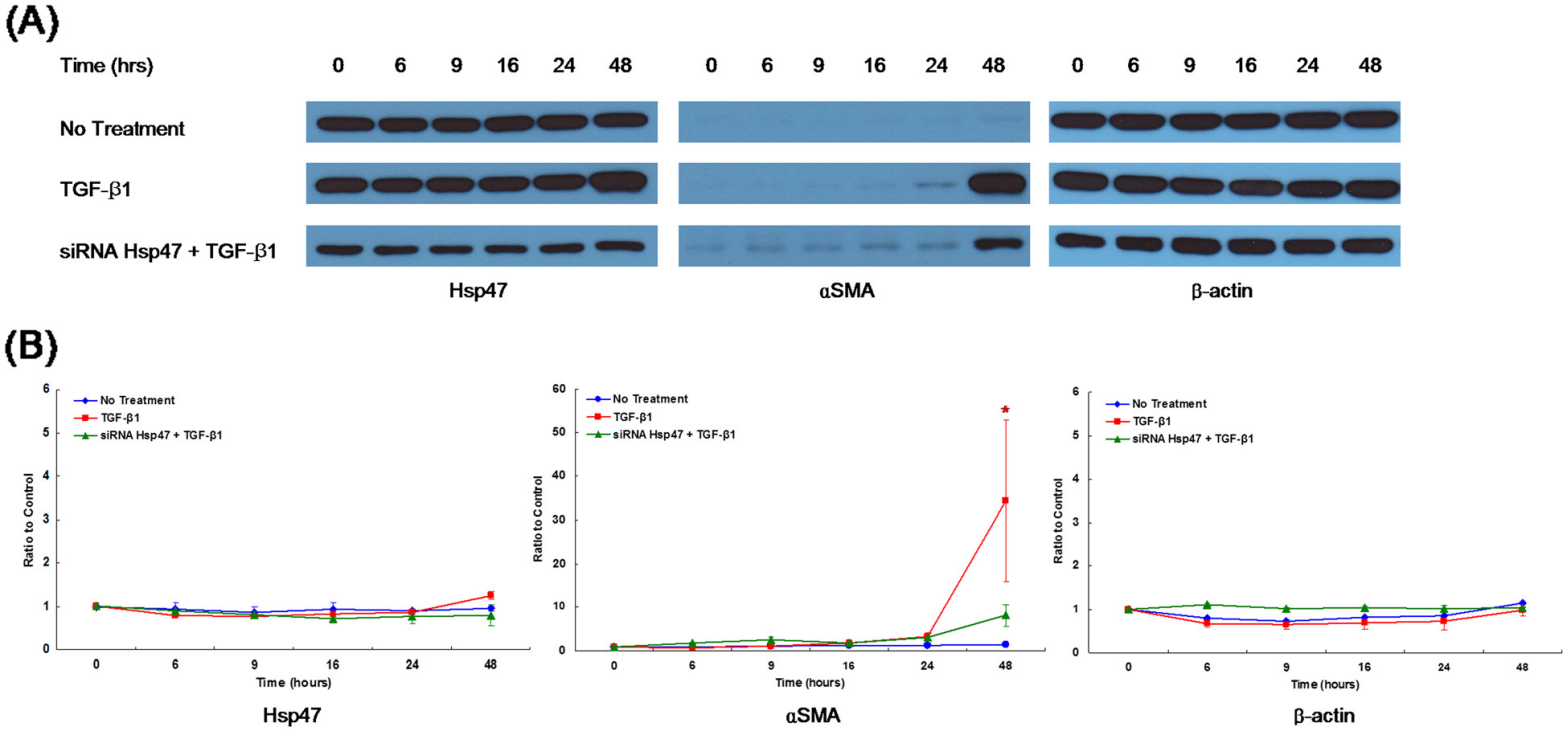
**Representative sequential bands of western immunoblots (A) and densitometric data (B) for heat shock protein 47 (Hsp47), α smooth muscle actin (αSMA), and β-actin.** Human Tenon’s fibroblasts were exposed to transforming growth factor-β1 (TGF-β1) for up to 48 hours with or without siRNAs targeting Hsp47. The densitometric data are expressed as the mean ± S.E.M. (n = 16 for each group) *p < 0.001.

The effects of TGF-β1 and siRNAs specific for Hsp47 on αSMA expression are shown using by time sequential western immunoblots (Figure 2). The expression of Hsp47 was not obviously changed by TGF-β1 treatment, whereas the expression of αSMA was significantly increased after 48 hours of TGF-β1 treatment (p = 0.011). When siRNA molecules targeting Hsp47 were introduced, the TGF-β1-induced αSMA expression was significantly attenuated, as was Hsp47 expression. After treatment with TGF-β1 for 48 hours, the ratio of αSMA immunobands relative to control was 1.52 ± 0.06, 34.39 ± 18.47, 8.18 ± 2.52 for the no treatment group, TGF-β1 treatment only group, and Hsp47 siRNA and TGF-β1 treatment group, respectively (p < 0.001). To eliminate the possible impact of transfection itself, scrambled siRNAs were also used ( Additional file 1). Presence of scrambled siRNAs did not make any apparent influence to the response of human Tenon’s fibroblasts caused by TGF-β1 treatment.

## Discussion

In most living tissues, fibrosis is an essential wound-healing response to noxious stimuli. If excessive, however, it can disrupt the normal function of affected organs. For subconjunctival fibrosis, TGF-β plays a crucial role and causes the transdifferentiation of Tenon’s fibroblasts to myofibroblasts and the subsequent production of extracellular matrix including collagen from the transdifferentiated myofibroblasts [2-5].

Hsp47 is a 47-kDa glycoprotein and specialized molecular chaperone in collagen biosynthesis. In the ER of any collagen-producing cells, Hsp47 binds to the triple helical procollagen and stabilizes its higher-order structure. It prevents the premature secretion of procollagens from the ER into the Golgi apparatus and concentrates them within the ER before finishing their maturation process [6-11]. The importance of Hsp47 in normal development was documented by Nagai et al. [10]. Using Hsp47 knockout mice, they showed that disruption of Hsp47 caused severe deficiencies in collagen maturation and organogenesis. The Hsp47 null embryos did not develop beyond 11.5 days postcoitus. In some animal models, siRNAs targeting Hsp47 effectively reduced experimental fibrosis [25,26].

The majority of previous studies have focused on the role of Hsp47 in collagen synthesis. However, in the present study using TGF-β-stimulated human Tenon’s fibroblasts *in vitro*, we noticed another putative role of Hsp47 in the fibrotic process. The silencing of Hsp47 with specific siRNA molecules significantly attenuated the expression of αSMA that was induced by TGF-β1 treatment. Our data imply that Hsp47 may be involved in the initial stage of fibrosis, the transdifferentiation of stressed fibroblasts to myofibroblasts, as well as the later stage of fibrosis, the collagen synthesis in already transdifferentiated myofibroblasts. Since the transdifferentiation of fibroblasts to myofibroblasts is thought to be a vital initial step for the whole fibrotic process, if Hsp47 directly or indirectly participates in this step, controlling its action might be a novel therapeutic strategy for patients suffering from excessive fibrosis. Actually, the Hsp47-associated transdifferentiation signaling may be not the major pathway in pathologic fibrotic processes. However, due to the fibrosis is the essential wound healing processes, its partial modification using Hsp47 pathway rather than complete suppression might be a better option to control the postoperative inflammation. To better understand the precise working mechanisms of Hsp47 in transdifferentiation of fibroblasts to myofibroblasts, the further investigations are needed.

Regarding expression of Hsp47 itself, our findings differ from previous reports that documented increased expression of Hsp47 in various extraocular fibrotic diseases [12-21]. Increased expression of Hsp47 was even reported in ocular cicatrical pemphigoid [22]. However, in this *in vitro* study using human Tenon’s fibroblasts, TGF-β1 increased the mRNA expression of Hsp47 but did not influence its protein expression. Data of immunofluorescence staining ( Additional file 2) support the results of western immunoblots. Though fibroblasts have been considered universal cells, they have different characteristics depending on their origin [27]. Their response to TGF- β1 and expression pattern of Hsp47 are able to be tissue-specific and/or disease-specific. In addition, a gap between mRNA and protein levels of Hsp47 is assumed to be caused by the post-transcriptional modulation.

## Conclusion

In summary, Hsp47, a well-known molecular chaperone specific for procollagen, appears to be related to the TGF-β1-induced transdifferentiation of human Tenon’s fibroblasts to myofibroblasts.

## Abbreviations

αSMA: α Smooth Muscle Actin; DMEM: Dulbecco’s Modified Eagle’s Medium; ER: Endoplasmic Reticulum; FBS: Fetal Bovine Serum; Hsp47: Heat shock protein 47; PVDF: Polyvinylidene Fluoride; SDS-PAGE: Sulfate Polyacrylamide Gel Electrphoresis; S.E.M.: Standard Error of the Mean; siRNA: small interfering RNA; TGF-β: Transforming Growth Factor-β.

## Competing interests

None of authors has a financial competing interest.

## Authors’ contributions

GJS, SH, and JBL designed the experiments; SH, KP, JHK, SHH carried out the molecular works; All authors were involved in interpretation of data and writing of manuscript. All authors read and approved the final manuscript.

## Pre-publication history

The pre-publication history for this paper can be accessed here:

http://www.biomedcentral.com/1471-2415/12/49/prepub

## Supplementary Material

Additional file 1 **Western immunoblots of the scrambled siRNA-introduced fibroblasts for heat shock protein 47 (Hsp47) (A), α smooth muscle actin (αSMA) (B), and β-actin (C).** siRNA Sc = scrambled siRNA; TGF-β1 = transforming growth factor-β1. Click here for file

Additional file 2 **Immunefluorescence of human Tenon’s fibroblasts for heat shock protein 47 (Hsp47).** (A) No treatment control; (B) TGF-β1 treatment. Nuclei were counterstained with 4',6-diamidino-2-phenylindole (DAPI). Click here for file
