# Supplementary figures and images for: Role of Heat Shock Protein 47 in Transdifferentiation of Human Tenon's Fibroblasts to Myofibroblasts
Source: BMC Ophthalmol. 2012 Sep 11;12:49. doi: 10.1186/1471-2415-12-49 (PMC3490793; doi:10.1186/1471-2415-12-49)

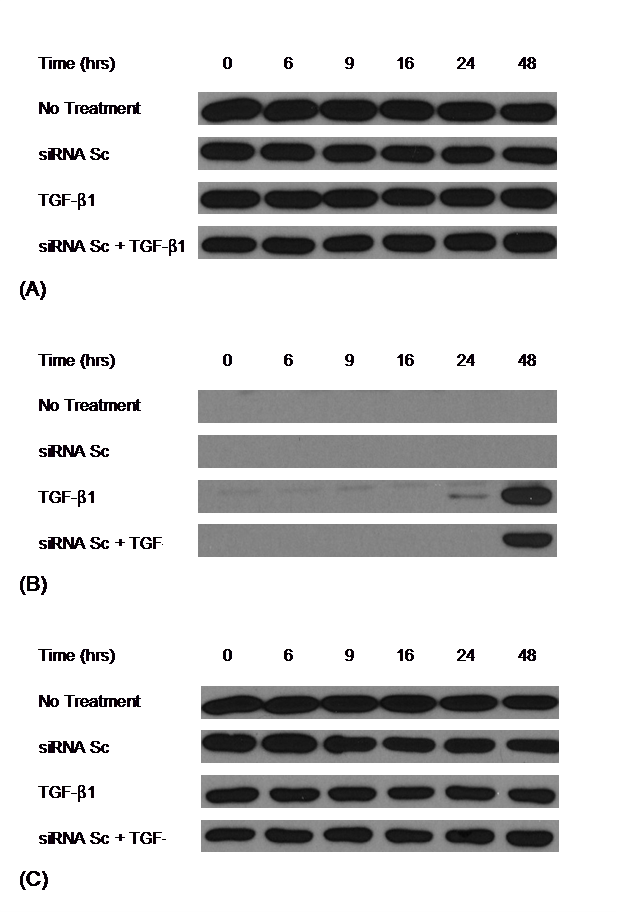

Supplement: Additional file 1 — Western immunoblots of the scrambled siRNA-introduced fibroblasts for heat shock protein 47 (Hsp47) (A), α smooth muscle actin (αSMA) (B), and β-actin (C). siRNA Sc = scrambled siRNA; TGF-β1 = transforming growth factor-β1. [file 1471-2415-12-49-S1.tiff]

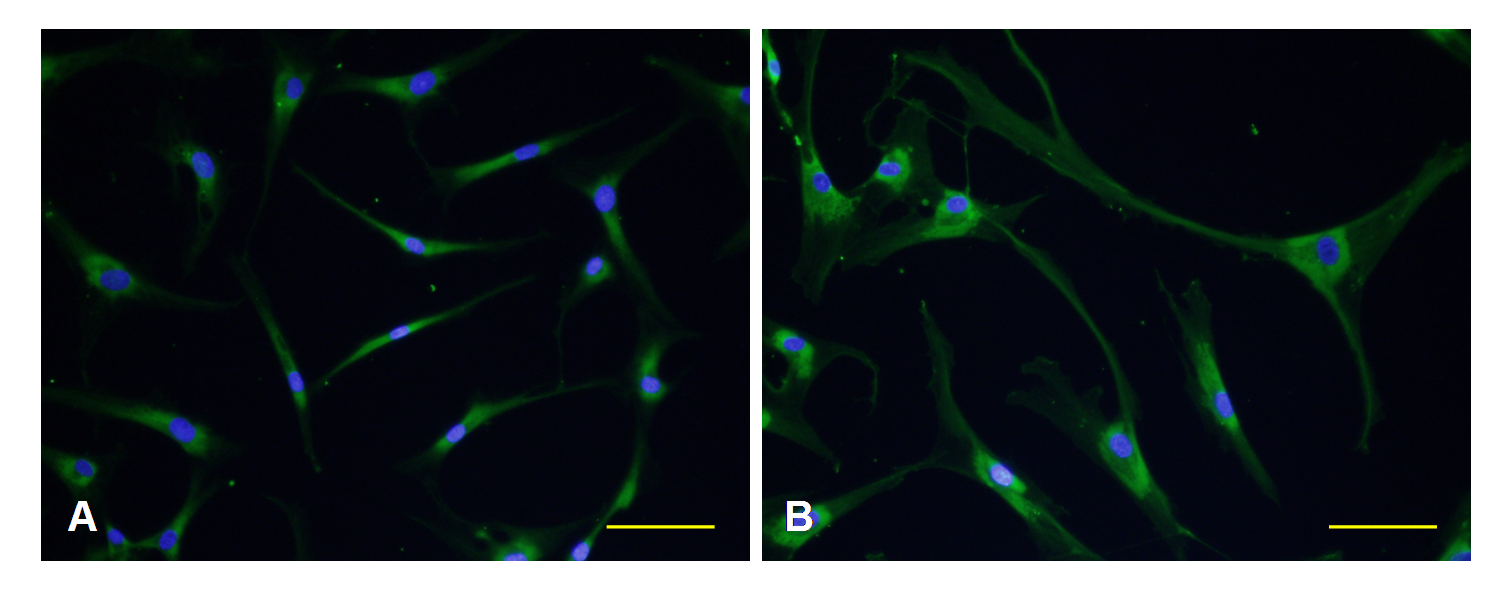

Supplement: Additional file 2 — Immunefluorescence of human Tenon’s fibroblasts for heat shock protein 47 (Hsp47). (A) No treatment control; (B) TGF-β1 treatment. Nuclei were counterstained with 4',6-diamidino-2-phenylindole (DAPI). [file 1471-2415-12-49-S2.tiff]
